# The Treatment of Puerperal Peritonitis

**Published:** 1894-10

**Authors:** Virgil O. Hardon

**Affiliations:** Atlanta, Ga.; Professor of Obstetrics and Gynecology in the Atlanta Medical College


### ATLANTA
### MEDICAL AND SURGICAL JOURNAL.

VOL. XI. OCTOBER, 1894. NO. 8.

EDITED BY
LUTHER B. GRANDY, M.D., AND MILLER B. HUTCHINS, M.D.,
WITH THE CO-OPERATION OF
H. V. M. MILLER, M.D., LL.D., VIRGIL O. HARDON, M.D.,
FLOYD W. McRAE, M.D., DUNBAR ROY, M.D.,
AND H. R. SLACK, M.D., PH.M.

### *ORIGINAL COMMUNICATIONS.*

### THE TREATMENT OF PUERPERAL PERITONITIS.

BY VIRGIL O. HARDON, M.D., ATLANTA, GA.,

Professor of Obstetrics and Gynecology in the Atlanta Medical College.

Puerperal peritonitis, in my estimation, is one of the most important disturbances with which the obstetrician has to deal. It fails to yield to treatment oftener than any other complication, and is unlike many other conditions which may arise that can be successfully managed by proper methods of treatment when seen early. Post partum hemorrhage and puerperal eclampsia, for instance, are serious accidents, but when seen early can be treated with confidence of a cure. This is not true of puerperal peritonitis, when it once becomes general. Garrigues says only a very small percentage of such cases recover.

There are two varieties of the disease according to extent of invasion, viz., local and general, and dependent upon this classification are the methods of treatment. General peritonitis in turn is

Synopsis of Remarks before Atlanta Obstetrical Society, July 12, 1894. See page 474.

divisible into two classes, viz., (1) that which has its origin primarily in a local peritonitis due to some injury to pelvic organs, or a retention of secundines; and (2)that developing not from an injury to pelvic tissues and not starting as a local trouble, but seemingly becoming general from the start.

Local peritonitis may depend upon disease of any pelvic organ, may arise from metritis, instrumentation, in fact any irritation to the uterus, disease of tubes or ovaries. Such cases usually run a definite course, pain, chill, elevation of temperature, acceleration of pulse, with imflammatory sequelæ; accumulations are more noticeable in Douglas's *cul-de-sac*, and many result in suppuration, with usual symptoms of pelvic abscess. Local inflammations starting as described do not always terminate in resolution, but may extend involving other tissues, causing temperature to run up to 104° to 105° F., pulse 130 to 160, tympanites, increase of pain, evidences of exhaustion, symptoms of collapse, and the patient sinks and dies. This makes up the usual history of a general peritonitis. There are two varieties according to products of inflammatory trouble, viz., those in which serum is thrown out, and those in which purulent material is also found.

TREATMENT.—I believe many of these cases when seen within from twenty-four to forty-eight hours after development can be aborted by free active purgation, using sulphate of magnesia, producing from twelve to fifteen watery actions within twenty-four hours.

If there be no pus in the tubes the peritonitis is controlled, pain relieved and patient benefited. It is my custom to give the salts daily, thus lessening the likelihood of an extension to entire peritoneum. If the disease progresses beyond two or three days, or lymph accumulates, the magnesia sulph. does not result in cure. In these cases opium is indicated if ever. If the disease becomes general, then the treatment is symptomatic. Here the continued use of salts is advisable, draining peritoneum through the bowels; at the same time exhaustion and high temperature must be attended to. With temperature not above 103° F. would be chary about using antipyretics, for by their use we mask one of our most important symptoms; if, however, it reaches 104° or 105° F., then antipyretics should be given to prevent the destructive effects of continued high temperature. Those cases not due to septic absorption beyond the resisting point may recover.

*Septic Peritonitis.*—In these cases salts has but little effect; it may temporarily relieve pain, but it soon returns. This may be due to the retention of decomposing products of conception, blood-clots, etc.; hence we should, when possible, remove the exciting cause. After washing out such a uterus, we sometimes have a marked amelioration of symptoms. Here a temperature of from 104° to 105° F. calls for antipyretics. Majority of these cases die. Have never seen a case of general septic peritonitis recover except those operated upon and washed out. Now, inasmuch as the majority of these cases die under the use of the remedies administered, the experiment of a few years ago (opening up and washing out) was a justifiable one. There have been a number of these cases, seemingly on the threshold of death, relieved by this means. Statistics show better results following the operative method in these cases than in any other form of treatment. A small incision, two to three inches in length, in median line is made, a tube inserted carrying hot water 110° to 115° F., thoroughly irrigating the entire abdomen. Such an operation is devoid of danger. Dr. Baldy sent queries to many obstetricians at home and abroad concerning operative measures, and in no case did any report death where nothing was removed from the abdomen. Those that recover do so rapidly. Every operation, like any other plan of treatment, must be regarded comparatively. Here it has lowered the mortality very appreciably; then why should it not be adopted more frequently?

The charge has been made that ambitious operators open the abdomen in such cases wantonly and from personal motives. If a favorable result was a certainty in all cases, such a charge might possess some element of plausibility. But unfortunately a considerable number will die in spite of the operation. The operator then has to brave the censure of the public and the criticism of the profession. If every man could know the writhing of spirit which the conscientious operator feels when he takes up the knife to operate in these desperate cases, there would be fewer insinuations about belly-rippers, etc. Nothing but the strongest sense of duty should cause any sensible man to undertake such an operation. I have operated on three cases, saving two. The one that died had pus in abdominal cavity. Those recovering were seen by conservative skillful men, not operators, who said the patients could not be saved by any other means. One of these cases when seen by me had pulse 148, temperature 104½° F., dark circles under eyes, pinched expression. Two of our best physicians said she could not survive many hours. She was operated upon and recovered. We recognize that opening the abdomen is a very serious operation, and should not be undertaken lightly. The time to operate is when we find the other methods fail to control symptoms. When in spite of salts, antipyretics, stimulants, etc., the patient grows worse, then it is time to operate.

